# Rapidly Progressing Primary Membranous Nephropathy in a Hispanic Male With Elevated Levels of Anti-phospholipase A2 Receptor Antibodies

**DOI:** 10.7759/cureus.15594

**Published:** 2021-06-11

**Authors:** Hamza Ashraf, Sotirios G Doukas, Pooja Gogia, Asim Khan

**Affiliations:** 1 Internal Medicine, Saint Peter’s University Hospital, New Brunswick , USA; 2 Department of Forensic Sciences and Laboratory of Toxicology, University of Crete, Medical School, Heraklion, GRC; 3 Internal Medicine, Saint Peter's University Hospital, New Brunswick, USA; 4 Internal Medicine, Saint Peter’s University Hospital, New Brunswick, USA

**Keywords:** nephrotic syndrome, membranous nephropathy, primary membranous nephropathy, anti-phospholipase a2 receptor antibodies, immunosuppression

## Abstract

A 49-year-old Hispanic male presented to the Emergency Department with progressively worsening swelling in his extremities for the past two months. Physical examination was significant for diffuse edema, with concomitant initial laboratory tests revealing hypoalbuminemia, hypercholesteremia, and proteinuria. A renal biopsy was performed, and the histopathology confirmed a diagnosis of membranous nephropathy through immunofluorescence and electron microscopy. Anti-phospholipase A2 receptor (anti-PLA2R) antibodies were detected on immunofluorescence, as well as high levels being discovered in the patient's serum, indicating a diagnosis of primary membranous nephropathy. The patient underwent adequate diuresis and was discharged. The patient presented six months later due to severe anasarca with laboratory tests indicating a rapid decline in renal function. He was then started on immunosuppressive therapy. Our rare case of a Hispanic male presenting with rapidly deteriorating renal function secondary to primary membranous nephropathy seeks to highlight the possibility of using anti-PLA2R antibodies as a marker for early initiation of immunosuppressive therapy as well as to encourage additional research on the course of disease progression in the Hispanic population.

## Introduction

Nephrotic syndrome is a type of renal disease characterized by an array of clinical and laboratory findings resulting from damage to the glomerular filtration barrier, leading to an excessive loss of protein in the urine. Nephrotic syndrome is defined by a urine protein excretion rate of greater than 3.5 grams per 24 hours, a serum albumin level of lesser than 3 grams per deciliter (g/dL), and hyperlipidemia. Clinical features of nephrotic syndrome include generalized edema, hypertension, and hypercoagulability [1,2]. Membranous nephropathy is a form of nephrotic syndrome that is characterized by a basement membrane that is diffusely thickened on light microscopy, subepithelial immune complex deposition causing a pathognomonic “spike and dome” appearance on electron microscopy, and immunofluorescence showing Immunoglobulin (Ig) G and C3 deposition [1]. The immune complexes in membranous nephropathy are thought to have been generated by antibodies formed against phospholipase A2 receptors or thrombospondin type-1 domain-containing 7A, which are antigens present on the foot process of podocytes [3,4]. Deposition of the immune complexes leads to localized inflammation and subsequent disruption and dislocation of the podocytes, thereby diminishing the integrity of the glomerular filtration barrier [2]. The presence of anti-PLA2R antibodies, which were found in a significantly high quantity in our patient, have been shown to be specific for primary or idiopathic membranous nephropathy [3]. Historically, membranous nephropathy is most commonly seen in Caucasian non-diabetic adults and, in contrast, is seen sparingly in the Hispanic population [5-7]. Here, we demonstrate a case of rapidly deteriorating renal function secondary to primary membranous nephropathy in a Hispanic male who was found to have an extraordinarily high level of anti-phospholipase A2 receptor antibodies.

## Case presentation

**Figure 1:**
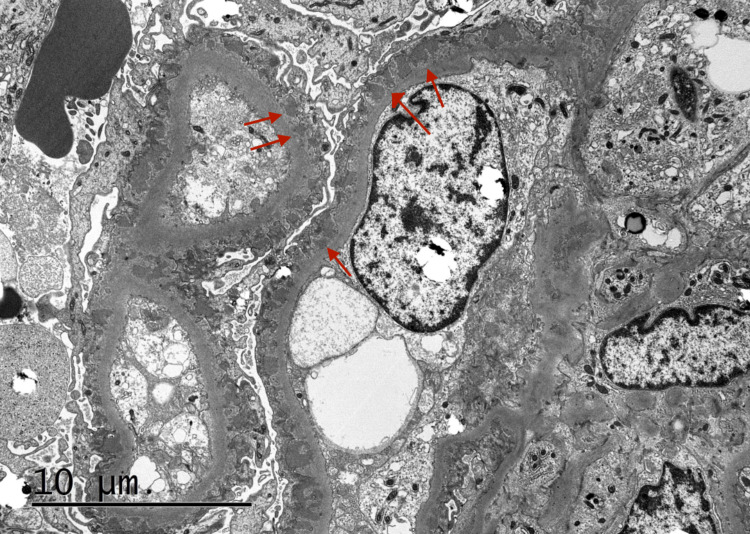
Electron microscopy revealing global sub-epithelial electron-dense deposits and foot process effacement There are characteristic “spikes and domes” present as a result of displaced podocytes lying down excess basement membrane.

**Figure 2:**
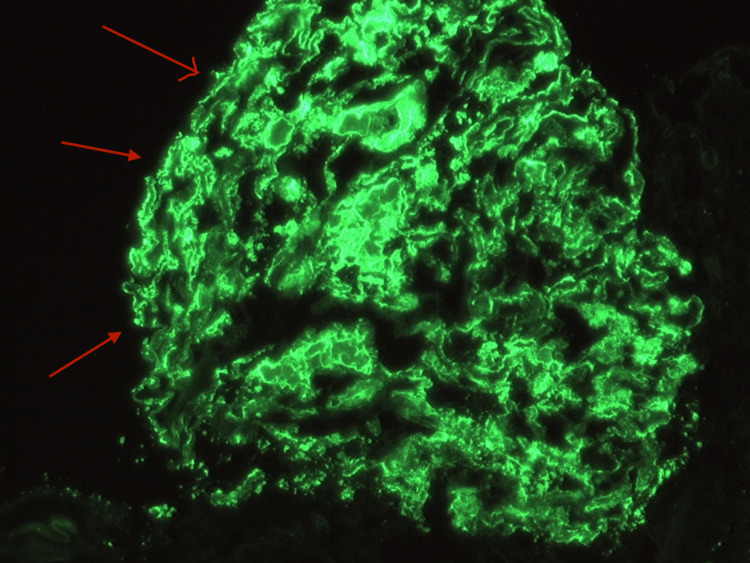
Immunofluorescence showing granular global glomerular capillary wall staining for IgG, C3, kappa, and lambda The granular nature of the immunofluorescent staining indicates immune complex deposition.

**Figure 3:**
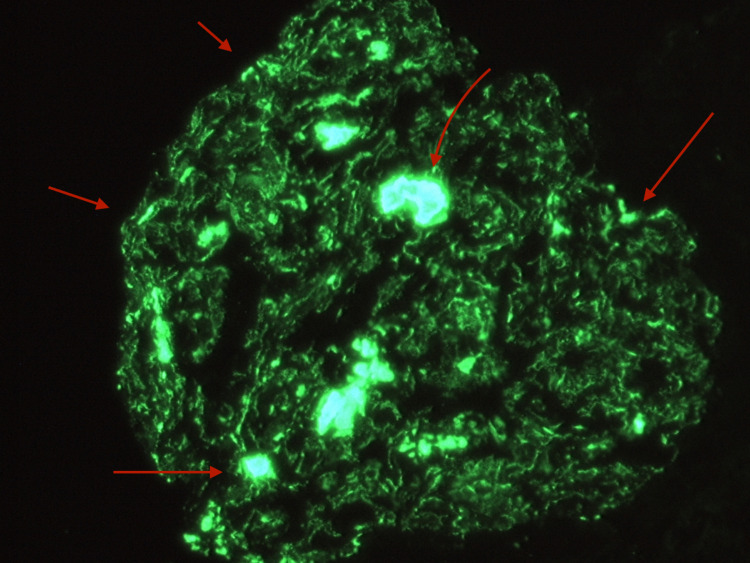
Immunofluorescence staining positive for specific IgG autoantibodies against PLA2R This suggests primary membranous nephropathy.

A 49-year-old Hispanic male from Honduras with no significant medical history presented to the Emergency Department due to progressively worsening swelling in his upper and lower extremities for the past two months. Vital signs on presentation consisted of a heart rate of 70 beats per minute, a blood pressure of 145/89 mmHg, a respiratory rate of 18 breaths per minute, a temperature of 98.5 degrees Fahrenheit, and an oxygen saturation of 98% on room air. Physical examination was significant for a moderately distended abdomen, periorbital edema, 2+ bilateral pitting edema in the lower extremities extending to the sacrum, and trace bilateral pitting edema in the hands and forearms. Laboratory tests revealed an albumin level of 0.9 g/dL via electrophoresis, a total cholesterol of 597 mg/dL with triglycerides of 441 mg/dL, and a urine protein greater than 1000 mg/dL. Serum blood urea nitrogen (BUN) and creatinine at the time of admission were 13 mg/dL and 0.87 mg/dL, respectively, with a glomerular filtration rate (GFR) of 93 ml/min/1.73 m2. Hemoglobin A1C, C3, C4, HIV, antinuclear antibodies, Hepatitis B, and Hepatitis C status were all within normal limits, which helped in excluding most secondary causes for the patient's presentation. After renal ultrasonography was found to be unremarkable, a renal biopsy was performed on the patient’s left kidney. Histopathology revealed dense subepithelial deposits graded as 3+ with associated foot process effacement, as shown in Figure 1. Immunofluorescence of the sample revealed 3+ glomerular capillary wall staining for IgG, 2+ staining for C3, 3+ staining for kappa, and 2+ for lambda, as shown in Figure 2. Anti-PLA2R receptor antibodies were also detected on immunofluorescence, as shown in Figure 3. Titers for anti-PLA2R antibodies were present in a quantity of 1499 relative units per milliliter (RU/mL) with a normal reference range of less than 19 RU/mL. The constellation of these findings solidified a diagnosis of primary membranous nephropathy. 

The patient’s hospital course was uncomplicated, consisting of five days of primarily receiving diuresis to obtain euvolemia. The patient was discharged on home medications of furosemide 20 mg twice a day, lisinopril 2.5 mg daily, aspirin 81 mg daily, and atorvastatin 20 mg daily. He was advised to follow a low sodium diet and to follow up with a nephrologist as an outpatient. He was not recommended immunosuppressive therapy at the time due to normal serum BUN and creatinine values. Despite compliance with discharge instructions, the patient was readmitted six months later due to severe generalized swelling and a bilateral transudative pleural effusion with laboratory findings showing a serum BUN of 44 mg/dL, creatinine of 2.12 mg/dL, and a GFR value of 44 ml/min/1.73 m^2, indicating a rapid decline in renal function. The patient was treated with heavy diuresis and was started on immunosuppression therapy with rituximab.

## Discussion

The incidence of membranous nephropathy is approximately eight to 10 per million. Membranous nephropathy is historically known to be most common in non-diabetic Caucasian adults [8]. However, membranous nephropathy is typically uncommon in the Hispanic population, especially without an underlying secondary cause [7,9]. Classically, the most common type of nephrotic syndrome in Hispanics is focal segmental glomerular sclerosis [9]. A study of glomerular diseases amongst the Hispanic population collected data from 1,040 renal biopsy specimens in children and adults with primary and secondary renal diseases. This study confirmed that the most frequent glomerulopathies in Hispanics are focal segmental glomerulosclerosis (27.7%), followed by lupus nephritis (17.8%), and immunoglobulin A nephropathy (9.4%). Out of the 1,040 biopsies obtained, only 88 showed evidence of membranous nephropathy, corroborating the fact that membranous nephropathy is rather rare in the Hispanic population [7].

The spontaneous remission of membranous nephropathy is not uncommon, and therefore, initial treatment is centered around supportive therapy unless specific criteria are met. These include a marked elevation in creatinine greater than 1.5 mg/dL, a decrease in eGFR of greater than 25 percent, and severe nephrotic syndrome with albumin levels of less than 2.5 g/dL [10]. Furthermore, studies have demonstrated that the presence of anti-PLA2R antibodies has a significant prognostic value, as lower levels of titers have been seen to correspond with both higher rates of spontaneous disease remission as well as a better response to immunosuppressive therapy [3]. Additionally, recent studies have suggested that there is merit in supplementing current proteinuria, eGFR, and BUN/creatinine driven treatment of membranous nephropathy with an individualized serology-based approach. The limitations regarding this approach stem from the fact that it gives less importance to the patient's clinical context as it pertains to assessing the current state of disease [11]. Nonetheless, the use of anti-PLA2R titers in making initial treatment decisions remains unclear. In the case presented, the rapidly declining kidney function of our patient could very well be associated with the presence of the high level of anti-PLA2R antibodies, which supports the idea that further extensive research needs to be done to explore their prognostic value as well as their value in guiding the initiation of targeted immunosuppressive therapy.

## Conclusions

This study presents a case of rapidly progressing primary membranous nephropathy in a Hispanic male with markedly elevated titers of anti-PLA2R antibodies. The findings presented underscore the possibility that the level of these titers could play a role in guiding the decision of whether or not to initiate early immunosuppressive therapy. Furthermore, because primary membranous nephropathy is rather infrequent in the Hispanic population, there is an incentive in pursuing additional research on assessing the course and risk for disease progression in this population. 

## References

[1] McCloskey O, Maxwell AP (2017). Diagnosis and management of nephrotic syndrome. Practitioner.

[2] Lai WL, Yeh TH, Chen PM (2015). Membranous nephropathy: a review on the pathogenesis, diagnosis, and treatment. J Formos Med Assoc.

[3] Beck LH Jr, Bonegio RG, Lambeau G (2009). M-type phospholipase A2 receptor as target antigen in idiopathic membranous nephropathy. N Engl J Med.

[4] Tomas NM, Beck LH Jr, Meyer-Schwesinger C (2014). Thrombospondin type-1 domain-containing 7A in idiopathic membranous nephropathy. N Engl J Med.

[5] Cattran DC, Brenchley PE (2017). Membranous nephropathy: integrating basic science into improved clinical management. Kidney Int.

[6] Couser WG (2017). Primary membranous nephropathy. Clin J Am Soc Nephrol.

[7] Arias LF, Henao J, Giraldo RD, Carvajal N, Rodelo J, Arbeláez M (2009). Glomerular diseases in a Hispanic population: review of a regional renal biopsy database. Sao Paulo Med J.

[8] Keri KC, Blumenthal S, Kulkarni V, Beck L, Chongkrairatanakul T (2019). Primary membranous nephropathy: comprehensive review and historical perspective. Postgrad Med J.

[9] Dragovic D, Rosenstock JL, Wahl SJ, Panagopoulos G, DeVita MV, Michelis MF (2005). Increasing incidence of focal segmental glomerulosclerosis and an examination of demographic patterns. Clin Nephrol.

[10] Fervenza FC, Appel GB, Barbour SJ (2019). Rituximab or cyclosporine in the treatment of membranous nephropathy. N Engl J Med.

[11] De Vriese AS, Glassock RJ, Nath KA, Sethi S, Fervenza FC (2017). A proposal for a serology-based approach to membranous nephropathy. J Am Soc Nephrol.

